# The Influence of Health-Promoting Leadership on Employees’ Positive Workplace Outcomes: The Mediating Role of Employability and the Moderating Role of Workplace Civility

**DOI:** 10.3390/ijerph192215300

**Published:** 2022-11-19

**Authors:** Chunyu Zhang, Liping Liu

**Affiliations:** School of Economics and Management, Guangxi Normal University, Guilin 541004, China

**Keywords:** health-promoting leadership, employability, workplace civility, positive workplace outcomes

## Abstract

The COVID-19 pandemic has severely accelerated the transformation and rapid organisational change in the workplace. The impact of the COVID-19 pandemic on the hotel industry will not fade in a short time, and the long-term coexistence with the COVID-19 pandemic pressure is a real dilemma for the hotel industry. The topic of How to create employee positive workplace outcomes (task performance and innovative work behaviour) during the COVID-19 pandemic has garnered increasing interest in both practical and academic fields. Leaders play a critical role in influencing employee workplace outcomes, yet few studies have explored the predicting role of health-promoting leadership. Drawing upon the conservation of resources (COR) theory, this study aims to examine the employability mediator effect and workplace civility as the moderator effect in the relationship between health-promoting leadership and employee-positive workplace outcomes (task performance and innovative work behaviour). We conducted a two-wave survey of 421 participants from the hotel industry in China and formulated a series of hypotheses that were tested with structural equation modelling. The results showed that health-promoting leadership has a significant positive effect on employees’ employability (*β* = 0.479, *p* < 0.001), task performance (*β* = 0.250, *p* < 0.001), and innovative work behaviour (*β* = 0.446, *p* < 0.001). Employability has a significant positive effect on task performance (*β* = 0.438, *p* < 0.001) and innovative work behaviour (*β* = 0.296, *p* < 0.001). This study makes certain contributions to the extant hotel industry employees’ positive workplace outcomes literature by attending to the healthy leadership styles that promote employability during the COVID-19 pandemic, and its novel point is to evaluate the workplace civility moderating effect between the above model. It also provides practical insight that mutual transformation in workplace relationships inspire those positive outcomes.

## 1. Introduction

As science and technology are changing rapidly and accelerating industrial transformation and enhancement, innovation has become a dynamic organic system with rich content and a complex hierarchical structure [1]. Exploring innovative behaviour will help enterprises modify innovation support policies that address competition attributable to globalisation, environmental uncertainty, and task complexity timely [2]. Accordingly, the past 20 years have seen increased attention to innovation as a scholarly research topic [3,4,5]. In this study, we focus on innovative behaviour at the employee level.

Previous research has demonstrated that distributed leadership influences innovation through employee wellbeing [6]. And technological innovation has a curvilinear effect on employees’ psychological wellbeing [7], based on the conservation of resources (COR) theory, which is an integrative stress theory that shows whether, at the perceptual or the objective level, the loss of existing resources and failure to obtain new resources leads to the individual stress response [8]. COR theory emphasises that individuals use key resources to cope with stressful situations in their current environment, or with potentially stressful situations in the future by investing their existing resources to obtain new resources [9]. With the development of organisational behaviour research, COR theory has emerged in a wider range of topics, including organisation membership [10,11], organisational citizenship behaviour [12], work engagement [13], innovation [14], the physical and mental health of employees [15], emotional labour [16], and work-family conflict [17]. Moreover, these studies also involve many common self-regulation and interpersonal interaction processes in the workplace [18]. With the knowledge economy development, more and more organisations emphasise flexible management and situational guidance, which encourages new perspectives in supportive leadership and a civil workplace climate [19].

At the level of situational factors, leaders expect to gain more development dividends through the transformation of hotels under COVID-19, so they will pay more attention to employees’ health awareness and workplace climate. Health will be a key influencing factor in the hospitality industry’s recovery after COVID-19 due to the residual fear associated with this pandemic and similar diseases [20]. With the COVID-19 pandemic, many people have begun to reconsider their work-lifestyles and focus on physical and mental well-being, considering this newly prominent consumer need, leading a healthy style could become a post-pandemic trend for hotels [21]. Accordingly, hotels should accommodate this public sentiment by formulating eco-friendly strategies and green practices in response to travellers’ concerns for the environment [22]. Health-promoting leadership studies have established the value of promoting health climate and positive workplace outcomes [23], but we do not understand its effective role in the COVID-19 pandemic, and still need to consider contextual factors. Health and productivity are often associated with other factors; for example, studies have shown that workplace relationships, physical and mental health, and productivity are inseparable, and employees influenced by leaders with greater health awareness will integrate innovation into their work, and exhibit more creative ideas and higher performance, employees may take a more positive attitude towards leaders’ actions, and, the positive workplace outcomes derived from individual promotion activities may be closely related to leadership health promotion, so we chose task performance and innovative work behaviour as positive workplace outcomes to be the dependent variables in the study.

At the level of individual factors, how employees deal with the transformation of the hotel industry caused by the COVID-19 pandemic depends on whether they focus on the positive results. Frontline employees can be viewed as the soul of a hotel as they represent the hotel and produce tangible and intangible services via direct interaction with customers. During COVID-19 pandemic, frontline employees become more important than ever because, without healthy employees, a hotel will be unable to instil trust and confidence in customers and restart activities that were frozen due to this pandemic [24]. It is worth noting that the COVID-19 pandemic has had a great impact on the hotel industry, which is increasingly paying attention to the creation of a healthy workplace and the marketing strategies to reconstruct health, such as digital detox plans and meditation plans. In order to better adapt to the new commercial mode, employees reshape the work content and relationship boundaries, employability is employee resources, which represent the orientation of aggressive motivation, and are helpful to task performance and innovative behaviour, which may be the inducement of positive workplace outcomes. The improvement of competence and job matching may increase motivation for employees’ task performance and innovative behaviour.

Further, studies of the roles of health-promoting leadership, employability, and workplace climate in moderating this relation are rare. Managers should maintain and develop workplace civility [25], which this study defines as practices and procedures intended to protect employees’ mental health, and proposes that it moderates the relation between job requirements and health outcomes [23]. However, we studied the more proximal roles of workplace civility and the way it is embedded in the leader-follower relationship. We expect that workplace civility will have a moderating effect on the relation between health-promoting leadership, employability, and positive workplace outcomes.

We propose a model that includes workplace factors, healthy leadership, employability, and positive workplace outcomes. Recognising that organisations function in society, economics suggest that together with organisational goals, there is a need for a healthy leadership style in the workplace that values employees’ health and a reasonable workload. Health-promoting leadership focuses on leaders developing and nurturing followers’ health, providing a fair workload, and a positive workplace climate, it is appropriate to research in the hotel industry that examines the effect of health-promoting leadership has on employees’ employability as positive workplace outcomes. We want to research whether health-promoting leadership plays a guiding role during the COVID-19 pandemic in the hotel industry; whether employability improves under the guidance of health-promoting leadership to adapt to the transformation of the COVID-19 pandemic; and whether workplace civility has a moderating effect in an environment of tense employee relations, layoff pressure, and heavy workload in the COVID-19 pandemic.

This study seeks to explore the role health-promoting leadership plays in positive workplace outcomes (task performance and innovative work behaviour), as well as explore the way employability mediates. We also aim to investigate the moderating role of workplace civility in the association between health-promoting leadership, employability, and positive workplace outcomes (task performance and innovative work behaviour). This study makes certain contributions to workplace outcomes with respect to the way leadership and workplace factors influence employees. First, we bring employability into leadership–workplace studies, and hereby emphasise its role in eliciting employee task performance and innovation. Second, we identify the specific role of health-promoting leadership, with the moderating role of workplace civility, not just in enhancing employee health, but also contributing to positive workplace outcomes through employability. Last, we conclude with a discussion of the way health-promoting leadership influences employees’ employability, and also contributes to positive workplace outcomes through the moderating effect of workplace civility. These findings are valuable, as they contribute to research on leadership practices and employees’ positive workplace outcomes. We integrated the COR theory, discussed the antecedents and consequences of employability, and the boundary effect between health-promoting leadership, employability, and positive workplace outcomes.

This study is constructed as follows. First, we review the literature on health-promoting leadership, employability, workplace civility, and positive workplace outcomes, and propose the research hypotheses. Second, we describe the research method and data collection processes. Third, we present the research results. Finally, we provide theoretical and practical implications.

## 2. Theoretical Background and Hypotheses

**Figure 1:**
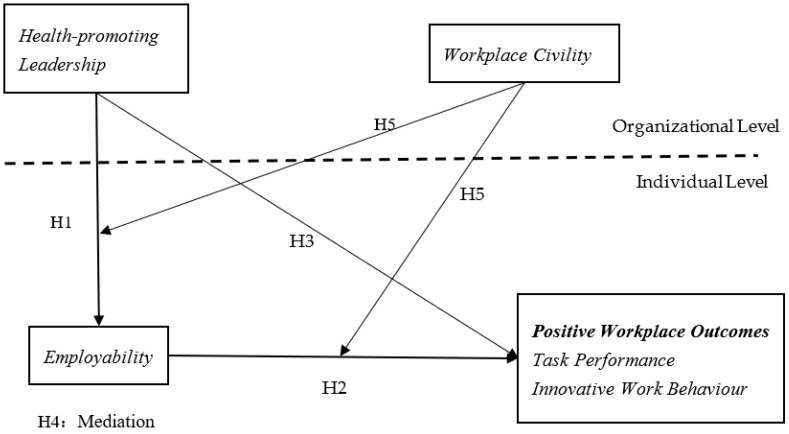
Research Framework.

COR theory emphasises that individuals use key resources to cope with stressful situations in their current environment, or with potentially stressful situations in the future by investing their existing resources to obtain new resources [9]. The COR theory can also explain why the COVID-19 pandemic affects employees’ emotions. This theory proposes that stress and EE occur when (1) individuals’ resources are threatened, (2) individuals’ resources are lost, or (3) when individuals fail to yield the anticipated re-turns following significant resource investment [8]. Halbesleben and Buckley [26] stated that emotional stress occurs not only when individuals have lost resources, but also when they become aware of threats to their resources. Therefore, during COVID-19, hotel industry employees’ perception of their resource loss, such as health and work conditions, can make them show a more positive attitude. Chen and Eyoun [27] also indicated that frontline restaurant employees need to spend both physical and psychological resources to cope with their fear of COVID-19, leading to resource loss to address anxiety at the workplace. Conceptually, employability captures what Hobfoll considered key resources, in which individuals acquire personal resources to ensure their individual survival in the workplace, and have complex skill systems for interpersonal communication, which is conducive to maintaining workplace relationships. In response to Hobfoll et al. [28], more attention should be given to the interaction among multiple resources and workplace factors’ influence. This study proposes that the interaction between health-promoting leadership, employability, and positive workplace outcomes follows the principle of the interaction of multiple resources among leadership types, workplace climate, and individual employees in the workplace (See Figure 1).

### 2.1. The Relation between Health-Promoting Leadership and Employability

COR theory, which emphasises the intrinsic and extrinsic motivational influences of social/workplace aspects that help employees achieve goals can explain the way the leadership and workplace climate support individuals’ personal resources [1]. Leadership may be seen as a supporting resource in the workplace [29]. Health-promoting leadership is a leadership style beneficial to organisations, as it focuses on valuing employees’ health, providing a fair workload, and engaging them in a healthy way. Further, it cultivates workplace civility [23].

Employability is regarded as a dynamic indicator, which changes as the organisational environment and employees’ work experience change [30], including the ability to maintain continuous employment and obtain re-employment [31,32].

The multi-dimensional health-promoting leadership adopted in this study has three dimensions: health consciousness, low workload, and control. Health-promoting leadership facilitates autonomy, employability, and satisfaction of employees’ needs, which echoes many employment elements, such as personal growth and leadership support, which are designed to satisfy employees’ needs in career development. Therefore, we suggest that health-promoting leadership, as a supportive leadership style, improves employees’ personal effective resources, as reflected by employability. Hence, we propose that:

**Hypothesis** **1.** *Health-promoting leadership is related positively to employees’ employability.*

### 2.2. The Relation between Employability and Positive Workplace Outcomes

Employability is the ability to obtain and maintain employment [31]. Highly employable individuals are more likely to identify employment opportunities and obtain jobs in the labour market [33]. With today’s uncertain and complex work environment, strong employability enhances individuals’ ability to manage uncertainties [34,35,36]. Accordingly, there is a positive relation between employability and employees’ wellbeing, and a negative relation between employability and job insecurity [37]. For example, social workers’ psychological wellbeing was found to be related positively to work engagement [38], which, in turn, affected their innovative work behaviour [39] and task performance [40].

In the rapidly changing employment environment, organisational leaders help employees by increasing their personal capacity reserve, updating knowledge, and enhancing development, while employees enhance their employability by participating in various forms of long-/short-term training that either their companies provide or they undertake voluntarily themselves [41]. Enhancing employability helps increase positive work outcomes such as task performance and innovative work behaviour [42]. For example, according to COR theory, taking into account both leaders’ and employees’ mutual interests in employability, employees seek access to training and platform resources to enhance their employability, and organisational leaders also want their employees to make better contributions to the organisation by providing them with training. Therefore, enhancing employability offers positive outcomes, such as the quality of job performance [43], to both employees and organisations. When employees are more employable, they will work harder to maintain such positive work outcomes as good task performance and innovative work behaviour [34,36]. Hence, we propose that:

**Hypothesis** **2.** *Employability is related positively to task performance and innovative work behaviour.*

### 2.3. The Relation between Health-Promoting Leadership and Positive Workplace Outcomes

Health-promoting leadership alleviates employees’ stress through various workplace strategies [44,45]. For example, employees’ self-efficacy and positive work outcomes are correlated positively with health-promoting leadership [46], and health-promoting leadership is associated negatively with job burnout and positively with job stress recovery [44,47]. Health-promoting leadership enriches the workplace environment by reducing employees’ workload, advocating for equitable resource distribution, and ensuring that employees’ efforts are rewarded. According to COR theory, when employees perceive acceptance and support in the work environment, psychological security is enhanced and leads to greater positive work outcomes [48]. Hence, we propose that:

**Hypothesis** **3.** *Health-promoting leadership is related positively to task performance and innovative work behaviour.*

### 2.4. The Mediating Role of Employability

The greater one’s career planning, skill development, and networking behaviours, the greater one’s employability [49]. We propose that an interactive workplace characterised by health-promoting leadership in a supportive atmosphere combined with civility creates a condition that motivates followers’ innovation actively. Employability promotes employees’ positive attitude toward workplace change [50,51], and is associated with the relation between occupational adaptability and job insecurity [52]. At the same time, in a civil workplace that promotes health, employees perceived sense of support and security enhances their employability, and also encourages them to innovate [53]. In this respect, employability serves as a mediator, and those who work under leadership that promotes health highly are more employable, have increased work skills, and demonstrate more positive work outcomes, i.e., task performance and innovative work behaviour. When employees perceive that they are less employable, it is expected that a leader’s potential to role model healthy, authentic behaviour, or encourage followers’ growth will be viewed as dishonest or inconsistent, reduce the relation to employability, and have no effect in promoting the positive work outcomes above. Hence, we propose the following:

**Hypothesis** **4.** *Employability mediates the relation between health-promoting leadership and task performance and innovative work behaviour.*

### 2.5. The Moderating Role of Workplace Civility

Workplace civility is correlated positively with collaboration [54] and affects employees’ work outcomes positively [55]. It creates a good working atmosphere [56], is conducive to colleagues exchanging and sharing information, and improves their engagement [57]. When individuals are treated with courtesy and respect, they will reciprocate [58], while when the workplace is less civil, ostracism may occur. This may be attributable to co-worker envy, which previous research has shown has a positive effect on ostracism and incivility in the workplace [59]. Further, employees who aspire to be more employable demonstrate a significantly lower preference for job security [60].

According to COR theory, the motivational shift from work resources to personal resources back to work resources results in a cycle of resource acquisition; for example, leaders who promote health support employees’ employability (Hypothesis 1). Employees with higher employability demonstrate good task performance and innovative behaviour (Hypothesis 2). Following this reasoning, we argue that workplace civility weakens the relation between health-promoting leadership and positive work outcomes through employability. Therefore, we propose the following:

**Hypothesis** **5.** *Workplace civility moderates the mediation effect between health-promoting leadership, employability, and task performance and innovative work behaviour, such that this mediation is weaker for individuals high in workplace civility than those low in workplace civility.*

## 3. Methods

### 3.1. Participants and Procedure

The participants in the questionnaire were employees in the hotel industry in China. The hotel industry is a typical labour-intensive industry, and service industry is an important pillar to support economic development; the COVID-19 pandemic has had an unprecedented impact on the development of the global hotel industry, and a large number of hotels have suffered from a sharp decline in occupancy and serious financial damage [61]. It is expected that the international tourism and accommodation industry will struggle to recover to the pre-epidemic level before 2023. Affected by the impact of the COVID-19 pandemic, Chinese hotels are facing the dilemma of high employee turnover rate, from a long-term perspective, retaining experienced labour resources is an important basis for the revitalisation of the hotel industry after the COVID-19 pandemic, and ensuring that hotel employees maintain a high level of positive work response is of great significance to promoting the sustainable development of the hotel industry [62]. This study mainly collects data from hotel front-line employees, such as the front office, housekeeping, food and beverage, maintenance, and recreation. Convenience sampling was adopted and the questionnaires were distributed from July 2021 to November 2021. To minimise common method variance, the research was conducted in two waves with online surveys [63]. We used WeChat, QQ, and emails to contact the employees. Those who were interested were sent a link that contained information about the study’s purpose and after the first survey, they were asked to provide their email addresses for the second survey. At Time 1, 537 participants responded to the first survey, which measured health-promoting leadership, employability, and control variables. One month later (Time 2), 445 of the original participants completed the second survey, which measured workplace civility, task performance, and innovative work behaviour. The final sample included 421 employees who completed both surveys, with a response rate of 78.4%.

### 3.2. Measures

The surveys measured the variables we investigated in this study: health-promoting leadership; workplace civility; employability; and positive workplace outcomes. Each item was rated on a 5-point Likert scale (1 = strongly disagree; 5 = strongly agree).

Health-promoting leadership (Time 1). To measure health-promoting leadership, we adopted a 9-item, three-dimensional scale that Jiménez, et al. [64] developed. A sample item is, “My leader will take care that the health of the employees is highly valued.” Its Cronbach’s alpha was 0.84.

Employability (Time 1). To measure employability, we adopted a 5-item, one-dimensional scale that Berntson and Marklund [65] developed. A sample item is, “I have a contact network that I can use to get a new (equivalent or better) job.” Its Cronbach’s alpha was 0.84.

Workplace civility (Time 2). A 13-item, three-dimensional scale that Di Fabio and Gori [66] was used to measure workplace civility. A sample item states: “My colleagues were able to express their values and their beliefs calmly to me.” In this study, the Cronbach’s alpha was 0.93.

Task performance (Time 2). This variable was measured using the 5-item scale that Koopmans, et al. [67] developed. A sample item is: “I was able to plan my work so that I finished it on time.” Its Cronbach’s alpha was 0.80.

Innovative work behaviour (Time 2). Innovative work behaviour was measured with a 10-item, one-dimensional scale De Jong and Den Hartog [68] developed. A sample item is: “I wonder how things can be improved.” Its Cronbach’s alpha was 0.91.

A structural equation model (SEM) was used to analyses the data. The ratio of the number of samples to the number of formal questions in the questionnaire was 10:1 [69]. There were 36 formal questions in the study, which meets the requirements for SEM analysis.

### 3.3. Ethical Considerations

The study was conducted in accordance with the Declaration of Helsinki, and approved by the Institutional Review Board of Guangxi Normal University. All participants were required to read and fill out the consent form before entering the research. Moreover, our surveys are anonymous, and employee’s personal information cannot be identified.

### 3.4. Descriptive Statistical Analyses

The participants’ characteristics are as follow. There were 238 (56.5%) male respondents and 183 (43.5%) females. More than half of the respondents held a bachelor’s degree 234 (55.6%), while 84 (20%) held a master’s degree. There were 190 (45.1%) respondents who had been employed for 5–10 years and 190 (24.2%) had been employed for 10–15 years, while 194 (46.1%) respondents had a monthly income of 5000–10,000 RMB, followed by 103 (24.5%) whose monthly income was 10,001–15,000 RMB.

## 4. Results

### 4.1. Common Method Variance (CMV)

This study examined CMV with SPSS v. 25 using Harman’s single factor analysis. The results showed that more than one factor was extracted, and the maximum degree to which a factor was explained was 39.613% (<50%) [63].

### 4.2. Confirmatory Factor Analysis

Confirmatory factor analysis (CFA) was adopted using AMOS v. 23 to examine the construct validity. The hypothesised five-factor CFA yielded an acceptable fit to our data: CMIN = 1691.68, df = 655, CMIN/DF = 2.58 (<3.0) [70]. Alternative models yielded a poorer fit to the data, as shown in Table 1.

### 4.3. Convergent Validity

Comprehensive reliability (CR) and average variance extraction (AVE) were used to evaluate the variables’ convergent validity [71,72]. As shown in Table 2, all factor loadings were significant. These results supported the discriminant validity of the research variables and excellent hypothesised measurement model.

### 4.4. Correlation and Discriminant Validity

A correlation analysis was performed with SPSS to calculate the relations among the variables, which showed a positive correlation. In addition, the square root of the AVE on the diagonal was greater than the correlation coefficient between the row and column. This demonstrated the variables’ discriminant validity (see Table 3).

### 4.5. Hypothesis Testing

Hypotheses 1–4 proposed in this study were assessed using an AMOS path analysis, and Table 4 shows the results. First, health-promoting leadership has a significant positive effect on employability (*β* = 0.479, *p* < 0.001), which provided support for H1. Next, the results supported H2, as employability is found to have a significant effect on task performance (*β* = 0.438, *p* < 0.001) and innovative work behaviour (*β* = 0.296, *p* < 0.001). The results for H3 show that health-promoting leadership was related positively to employees’ task performance (*β* = 0.250, *p* < 0.001) and innovative work behaviour (*β* = 0.446, *p* < 0.001). This provided support for H3. Finally, H4 proposes that employability mediates the relation between health-promoting leadership and task performance and innovative work behaviour. To test this effect, it is necessary to compare health-promoting leadership’s total effect on both and the indirect effect between them.

As we noted previously, health-promoting leadership’s total effect on task performance and innovative work behaviour is significant, such that there is a direct relation between them. The coefficient of the relation between health-promoting leadership and the two increases after the variability in differential employability is controlled. Bootstrap sampling in the indirect effect model shows that the indirect effect of health-promoting leadership on task performance and innovative work behaviour through differential employability is statistically significant. Given the two conditions Preacher and Hayes stipulated [54], our mediation hypothesis (H4) for employability is supported.

**Figure 2:**
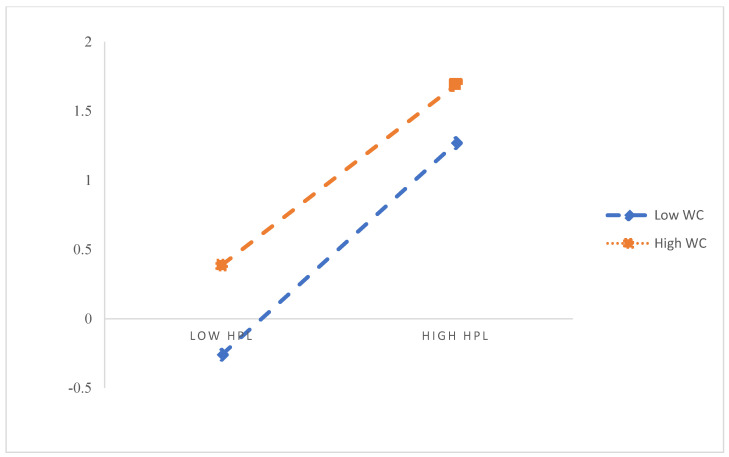
The moderating role of WC in the relationship between HPL and Ey.

**Figure 3:**
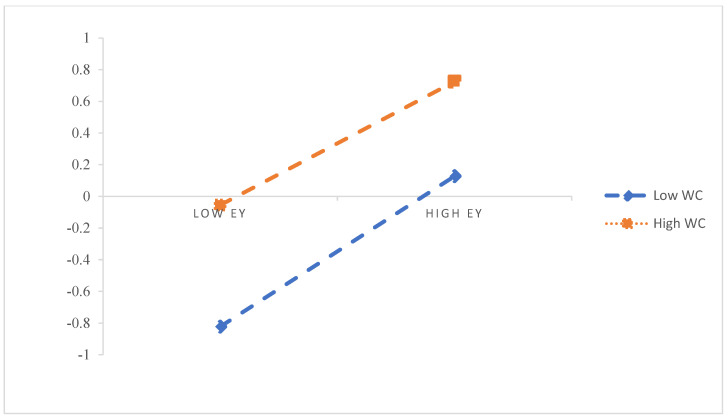
The moderating role of WC in the relationship between Ey and IWB.

H5 proposes that workplace civility has a moderating effect on the relation between employability, task performance, and innovative work behaviour. Mode 58 in the SPSS macro Hayes [73] compiled was adopted in this study. Table 5 and Table 6 show that the latent interaction variable, “HPL * WC”, has a significant path coefficient (*β* = −0.11, *t* = −2.44, *p* < 0. 05), indicating that workplace civility has a moderating effect on health-promoting leadership’s role in employability. Further, the results show that the latent interaction variable, “Ey * WC”, has a significant path coefficient (*β* = −0.10, *t* = −2.25, *p* < 0.05), indicating that workplace civility plays a moderating role in health-promoting leadership’s effect on employability and employability on innovative work behaviour. Accordingly, the research supports H5 in part. Figure 2 and Figure 3 present the moderating effect of workplace civility.

Further, a simple slope analysis (see Figure 2) shows that HPL has a significant positive effect on Ey (*β* = 0.43, *t* = 7.67, *p* < 0.001) with a lower WC level (M − 1SD). With a higher WC level (M + 1SD), HPL has a significant positive effect on Ey (*β* = 0.27, *t* = 3.73, *p* < 0. 01), but its prediction effect is weak. This showed that HPL’s predictive effect on Ey decreases gradually as the WC level increases. As can be seen in Figure 3, Ey has a significant positive effect on IWB with a low WC level (M − 1SD), (*β* = 0.23, *t* = 5.61, *p* < 0. 001). With a higher WC level (M + 1SD), Ey still has a significant positive effect on IWB (*β* = 0.10, *t* = 1.97, *p* < 0.05), but its predictive effect is weakened. The results showed that Ey’s predictive effect on IWB decreased gradually as the WC level increased.

## 5. Discussion

This study conducted two waves of surveys of hotel industry employees in China. As prior literature has touched upon, the role of health-promoting leadership in positive workplace outcomes; however, it still lacks a comprehensive understanding of the mediating and moderating mechanisms through which health-promoting leadership promotes positive workplace outcomes. To fill these gaps, we, in accordance with COR, examined the mechanism and boundary of the effect of health-promoting leadership and workplace civility on positive workplace outcomes. We proposed five hypotheses. Except for Hypothesis 5, which was partly supported, all our hypotheses have been supported.

The positive workplace outcomes of hotel employees in normal situations have been generally valued by the academic community, while research on the positive workplace outcomes of employees in major crises such as the COVID-19 pandemic is still rare. The impact of the COVID-19 pandemic on the hotel industry will not fade in a short time, and the long-term coexistence with the COVID-19 pandemic pressure is a real dilemma for the hotel industry [74]. Under the impact of the “abnormal” crisis of the COVID-19 pandemic, internal and external factors such as the development opportunities of the hotel industry, the market operating environment, and the physical and mental conditions of employees will change significantly [75]. So, it is an urgent research topic with important practical significance in the hotel industry.

Health-promoting leadership is found to affect employees’ positive workplace outcomes positively, consistent with previous studies [23]. Research on the effects of health-promoting leadership in Asian cultures is still scarce and inconclusive among various leadership styles [76]. Thus, the results of this analysis contribute to our existing knowledge about the effectiveness of health-promoting leadership in Asian hierarchical cultures. Affected by the COVID-19 pandemic, hotel employees are faced with a high degree of uncertainty. One of the most important psychological problems caused by the COVID-19 pandemic is the sense of uncertainty. A high level of health-promoting leadership in the hotel workplace means that the hotel invests a lot in employees, may care more about their work, life and physical and mental health, can find out the difficulties faced by employees due to the outbreak of the COVID-19 pandemic in a more timely manner, and make efforts to improve the current situation of employees.

The exploration of a positive association between health-promoting leadership behaviour and employability with respect to additional workplace resources and work skills corroborates previous research that has shown that health-promoting leadership plays an essential part in motivating employees to engage in proactive behaviour. This study offers evidence that health-promoting leadership relates positively to employees’ employability, and the manner in which employees perceive and experience health-promoting leaders in the workplace has implications for the way leaders care about their health, workload, and control, as indicated by the strong positive association between health-promoting leadership and employability. People-oriented leadership styles are a context-specific job resource that allows employees autonomy and opportunities to train themselves, which supports their personal ability to become more employable [77]. Affected by the epidemic, employees face higher environmental uncertainty, as they need to learn more knowledge about epidemic prevention measures, digital health, and digital detoxification plan. At this time, the health and safety hotel climate become an important source of information for them to predict the future be-haviour of the hotel, making them more likely to believe that the uncertainty caused by the epidemic is only temporary and will be improved over time [78]. The hotel will support themselves to overcome the difficulties. Therefore, Employees may have higher positive workplace outcomes (task performance and innovative work behaviour).

Finally, we examined workplace civility’s moderating effect between health-promoting leadership, employability, and task performance and innovative work behaviour. Our findings suggest that workplace civility moderates this relation negatively. Unlike prior research, workplace civility rarely serves as a moderating variable, while as a workplace climate, it affects the strength of the relation above. Workplace civility creates a good working climate [56], is conducive to colleagues exchanging and sharing information during the COVID-19 pandemic, and improves their innovative work behaviour [57]. With the support of a civilised workplace, hotel employees are establishing a positive personal appearance, reducing the risk of resource damage. In addition, relatively free working environment and massive digital information activated their innovative work behaviour and employability.

### 5.1. Theoretical Implications

This study expands the research on employability, enriches the theoretical and empirical basis for the cause and effect of employability, and complements existing theories in the following areas.

Employability is regarded as a dynamic indicator, as personal resources, which continue to change with the change in organisational environment and work experience, promote employees’ positive attitude toward workplace change [50]. Prior studies on employability have focused on students [79], and there has been little research on employees. Health-promoting leadership concerns consciousness of employees’ health, low workload, and control of work resources, and this definition echoes many employment elements, such as personal growth, leadership support, and positive relationships, which facilitate employees’ positive workplace outcomes [23].

Previous research has revealed that managerial support stimulates sustainable employability directly [80]. Further, employability mediates the relation between job characteristics as well as managerial support and employees’ employment opportunities [81]. The results of this study support the positive relation between health-promoting leadership and positive workplace outcomes such as task performance and innovative work behaviour, as well as employability’s significant mediating role. Thus, this study establishes a comprehensive framework that illustrates employability’s complex effects on antecedents and outcomes.

Moreover, previous research has found that health-promoting leadership affects job engagement positively [82]. Accordingly, we explored the way health-promoting leadership leads to positive workplace outcomes, and find results consistent with previous research. We also discuss workplace civility’s moderating role, which is a new topic in workplace climate that acts, in particular, on the relation between health-promoting leadership, employability, and positive workplace outcomes. This is a new exploration of organisational behaviour that complements the discussion of resources in COR theory. These results echo previous research finding that servant leadership had a stronger relation with eudaimonia wellbeing when the workplace civility climate was high [1].

### 5.2. Practical Implications

This study provides a strong avenue and contextual condition under which organisations affect and develop employees’ employability, with a concentration on health-promoting leadership in a civil workplace context. For the mutual benefit of both organisational performance and employees’ employability, it is recommended to identify and train leaders in ways to promote health, with an emphasis on empowering employees, helping them use their skills, and reducing their workload to achieve healthy behaviour. Both leadership and HR policies and practices may indeed be promoting a civil work environment that discourages unfriendly, aggressive behaviour actively and encourages polite interaction among employees. This study provides practical insight into the way a psychosocially safe atmosphere could be implemented in an organisation. Firstly, leaders should promote friendly cooperation among colleagues, which improves workplace civility and enhances employees’ positive outcomes. In addition, leaders should also attach importance to workplace ostracism, whether it is visible or not, and attempt to establish a harmonious and supportive workplace instead. Further, employees should be encouraged to integrate into the group to alleviate occupational tension, and psychological service departments can be set up in the organisation if necessary. Secondly, enterprises should pay attention to the effectiveness of employee training and respect their employees’ needs for labour and work skills training. Enterprises can carry out training programs according to their own characteristics, identify various career channels to do so, and the possibility of mutual transformation in workplace relationships that inspire positive outcomes.

## 6. Conclusions

Based on COR theory, this study emphasises the value of health-promoting leadership on employability and positive workplace outcomes. The conclusions are summarised as follows: Health-promoting leadership has a significant positive effect on employability, and employability is also helpful to improve positive workplace outcomes (task performance and innovative work behaviour). In addition, employability mediates the effects of health-promoting leadership on task performance and innovative work behaviour. Further, workplace civility plays a moderating role in health-promoting leadership’s effect on employability and employability on innovative work behaviour.

## 7. Limitations and Future Directions

Nevertheless, like all research, this study has some limitations. Firstly, as this study mainly focuses on the front-line employees of hotels affected by the COVID-19 pandemic, the perception of Chinese hotel employees on health-promoting leadership and positive workplace outcomes may have different characteristics in different regions, the general applicability of these findings should be examined in different specifications hotels in future research to compare the cultural differences with those of the employees of Chinese companies. Secondly, questionnaire recovery was in November 2021 which was during the COVID-19 pandemic, and hotel employees’ perception of health-promoting leadership was changing. In order to further explore the hotel employees’ positive workplace outcomes during the COVID-19 pandemic, future research can be within different regions to analyse the regional differences in the positive work results of hotel employees in the risk environment of the epidemic and, at the same time, to conduct follow-up research at different stages of the epidemic development, to analyse the characteristics of changes in the positive work results of hotel employees. In addition, this study only discusses three dimensions of health-promoting leadership: health consciousness, low workload, and control. In future research, cultural climate, hotel types, and other conditional variables can be added to the model to further enrich the model explanatory. Moreover, self-assessment methods are used to measure employability, which may bias the data’s objectivity. In future research, self-report, leader–employee matching data, and other reporting methods could be used to improve data objectivity.

## Figures and Tables

**Table 1:** Model fit.

	CMIN/df	GFI	IFI	TLI	CFI	RMSEA	RMR
Hypothesised five-factor model: HPL, Ey, WC, TP, IWB	2.583	0.810	0.908	0.901	0.908	0.061	0.054
Hypothesised four-factor model: HPL, Ey, WC, TP + IWB	3.891	0.703	0.831	0.819	0.830	0.083	0.064
Three-factor model: HPL+ Ey, WC, TP + IWB	5.205	0.618	0.753	0.737	0.752	0.100	0.081
Two-factor model: HPL+ Ey + WC, TP + IWB	7.534	0.461	0.615	0.591	0.613	0.125	0.103
Single-factor model: HPL+ Ey + WC + TP + IWB	8.248	0.441	0.572	0.546	0.571	0.131	0.102

Notes: HPL, Health-promoting Leadership; Ey, Employability; WC, Workplace Civility; TP, Task Performance; IWB, Innovative Work Behaviour.

**Table 2:** Confirmatory factor analysis summary.

	Items	Factor Loading	CR	AVE		Items	Factor Loading	CR	AVE
Health-promoting Leadership	HPL1	0.684	0.929	0.596	Workplace Civility	WC1	0.727	0.946	0.575
	HPL2	0.845				WC2	0.715		
	HPL3	0.857				WC3	0.789		
	LL1	0.835				WC4	0.817		
	LL2	0.714				WC5	0.774		
	LL3	0.873				WC6	0.767		
	Cl1	0.716				WC7	0.741		
	Cl2	0.746				WC8	0.834		
	Cl3	0.639				WC9	0.670		
Employability	Ey1	0.784	0.892	0.623		WC10	0.685		
	Ey2	0.810				WC11	0.762		
	Ey3	0.791				WC12	0.797		
	Ey4	0.803				WC13	0.765		
	Ey5	0.759			Innovative Work Behaviour	IWB1	0.656	0.895	0.590
Task Performance	TP1	0.713	0.892	0.625		IWB2	0.724		
	TP2	0.776				IWB3	0.718		
	TP3	0.850				IWB4	0.889		
	TP4	0.895				IWB5	0.823		
	TP5	0.701				IWB6	0.777		

**Table 3:** Summary of Correlation Analysis.

	M	SD	Gender	Age	Education Level	Working Years	Monthly Income	HPL	WC	Ey	TP	IWB
Gender	1.57	0.496	1									
Age	2.40	0.726	−0.136 **	1								
Education Level	2.91	0.794	0.015	0.133 **	1							
Working Years	2.51	0.975	−0.128 **	0.560 **	0.152 **	1						
Monthly Income	2.44	0.985	0.022	0.302 **	0.319 **	0.382 **	1					
HPL	3.763	0.766	0.015	0.211 **	0.128 **	0.120 *	0.048	0.772				
WC	3.652	0.733	0.021	0.130 **	0.117 *	0.064	0.060	0.556 **	0.758			
Ey	3.729	0.775	−0.063	0.128 **	0.215 **	0.112 *	0.131 **	0.453 **	0.347 **	0.789		
TP	3.626	0.815	0.037	0.090	0.125 *	0.122 *	0.038	0.476 **	0.401 **	0.501 **	0.791	
IWB	3.717	0.720	−0.028	0.155**	0.191 **	0.137 **	0.052	0.594 **	0.614 **	0.465 **	0.445 **	0.768

Notes: * *p* < 0.05. ** *p* < 0.01. The diagonal is the AVE square root of the corresponding construct.

**Table 4:** The effect of health-promoting leadership on positive workplace outcomes through employability.

		Coefficient	Confidence Intervals Bias Corrected	
			Lower Confidence Level	Upper Confidence Level
Total effects	HPL → TP	0.460 **	0.344	0.559
	HPL → IWB	0.587 **	0.480	0.683
Direct effects	HPL → TP	0.250 ***	0.109	0.369
	HPL → IWB	0.446 ***	0.319	0.536
	HPL → Ey	0.479 ***	0.352	0.587
	Ey → TP	0.438 ***	0.313	0.554
	Ey → IWB	0.296 ***	0.204	0.416
Indirect effects	HPL →Ey → TP	0.142 **	0.091	0.213
	HPL → Ey → IWB	0.210 **	0.183	0.312

Notes: ** *p* < 0.01. *** *p* < 0.001. HPL, Health-promoting Leadership; Ey, Employability; TP, Task Performance; IWB, Innovative Work Behaviour.

**Table 5:** Moderated mediation model testing.

	Model 1 (Ey)			Model 2 (TP)			Model 3 (IWB)		
	β	SE	t	β	SE	t	β	SE	t
constant	0.504	0.550	0.917	−0.121	0.594	−0.204	−0.348	0.457	−0.760
HPL	0.764	0.166	4.617 ***	0.240	0.054	4.407 ***	0.259	0.042	6.200 ***
WC	0.534	0.168	3.170 **	0.436	0.185	2.360 *	0.682	0.142	4.800 ***
Ey				0.611	0.173	3.527 ***	0.480	0.130	3.566 **
HPL * WC	−0.114	0.047	−2.438 *				−0.114	0.047	−2.438 *
Ey * WC				−0.074	0.049	−1.516	−0.09	0.040	−2.25 *
R^2^	0.229			0.348			0.507		
F	41.384			55.447			106.952		

Notes: * *p* < 0.05. ** *p* < 0.01. *** *p* < 0.001.

**Table 6:** Moderating effects at different levels of WC in Indirect effects (HPL → Ey → IWB).

	WC	Effect	Boot SE	Boot LLCI	Boot ULCI
effe1	(M − 1SD)	0.098	0.025	0.052	0.149
effe2	(M)	0.057	0.017	0.028	0.092
effe3	(M + 1SD)	0.027	0.017	0.000	0.065
effe2-effe1	(M − 1SD)	−0.041	0.017	−0.077	−0.009
effe3-effe1	(M)	−0.071	0.027	−0.125	−0.018
effe3-effe1	(M + 1SD)	−0.030	0.011	−0.050	−0.009

## Data Availability

There is no public access to all data generated or analysed during this study, in order to preserve the privacy of the identities of the individuals. The dataset that supports the conclusions is available to the corresponding author upon request.
